# Development of a research mentorship guide and consensus statement for low- and middle-income countries: Results of a modified Delphi process

**DOI:** 10.1371/journal.pone.0291816

**Published:** 2023-10-25

**Authors:** Fiona Kennedy, Annabel Steiner, Joseph D. Tucker, Mirgissa Kaba, Alemseged Abdissa, Noah Fongwen, Eneyi E. Kpokiri

**Affiliations:** 1 Department of Clinical Research, London School of Hygiene and Tropical Medicine, London, United Kingdom; 2 Institute of Global Health and Infectious Diseases, University of North Carolina at Chapel Hill, Chapel Hill, North Carolina, United States of America; 3 School of Public Health, Addis Ababa University, Addis Ababa, Ethiopia; 4 Armauer Hansen Research Institute, Addis Ababa, Ethiopia; Lahore Medical and Dental College, PAKISTAN

## Abstract

**Background:**

Institutional research mentorship is a form of mentorship whereby institutions foster mentor-mentee relationships. Research mentorship improves research effectiveness and supports relationships. However, resources are needed in order to institutionalize research mentorship tailored to low- and middle- income countries (LMICs). The aim of this study was to develop a consensus document on institutionalizing research mentorship through a modified Delphi process as part of the practical guide development process.

**Methods:**

This study used a two-round modified Delphi process, which is an iterative, structured approach of consensus decision making. Each participant was asked about a series of items related to research mentorship using Likert scale questions. Agreement for each item was pre-defined as ≥80% of participants rating the item as “agree” or “strongly agree.” The items that reached agreement, were then discussed during round two at an in-person conference in Ethiopia. A separate group of individuals only participated virtually. For the final consensus survey, response rates and commenting rates (participants who wrote two or more comments) were compared among conference and non-conference participants.

**Results:**

The Delphi process led to the inception of three main themes in terms of developing research mentorship: leveraging existing resources, measuring and evaluating institutional mentorship, and encouraging a research mentorship life cycle. During the virtual first round, 59% (36/61) participants who were emailed completed the survey. In the second round, conference participants had a response rate of 79% (11/14) compared to non-conference participants with a response rate of 45% (21/47). Conference participants had a 100% (11/11) commenting rate whereas non-conference participants had a 38% (8/21) commenting rate. This study achieved consensus in both survey rounds for all 35 items on the consensus document.

**Conclusions:**

The data suggest that an in-person conference may increase participant engagement. The consensus developed through a modified Delphi method directly informed a practical guide on institutionalizing research mentorship in LMICs.

## Background

Before Odysseus leaves on his journey in Homer’s ancient poem, The Odyssey, he entrusts his son Telemachus’ welfare to his closest and oldest friend, Mentor. As guardian, friend, sponsor, and counselor to Telemachus, Mentor then defines the oldest documented practice of mentorship which continues to play a critical role in society today [1]. Mentorship may be described most generally as having the foundational elements of knowledge sharing and learning promotion throughout its many forms [2]. Research mentorship can help mentees improve scientific and grant-writing skills, promote career development, and help the mentee transition to scientific independence [3]. Although mentorship often describes a specific relationship between two people, institutionalizing research mentorship is important [4].

Institutionalizing research mentorship nurtures research capacity in organizations (e.g., universities, professional associations, and research institutes) to improve research effectiveness and health equity [5]. Comprehensive research mentorship resources such as toolkits and practical guides can promote effective institutional research mentorship [6]. However, most of the available resources for research mentorship are tailored for high-income countries (HICs) [7]. Research institutions in HICs may have a longer tradition of supporting research mentorship and have greater institutional resources to sustain mentorship compared to LMICs [8].

Existing mentorship resources focus on the mentor-mentee relationship [8]. They often do not take into consideration the many forms of mentorship and the wider institutional factors (such as existing resources, cultural norms) that contribute to research mentorship [8]. There is a need for the development of an institutional research mentorship guide for LMIC researchers [9]. This paper answers the research question: Using a Modified Delphi process, how can we develop and build consensus for items within a practical guide focused on enhancing institutional research mentorship in LMICs? The Delphi method is a consensus-building process which is widely used by researchers to achieve consensus in diverse fields. The Delphi method is based off of the assumption that aggregated group opinions are more accurate than individual opinions [10]. The objective of our research is to build consensus on a core set of items on enhancing and institutionalizing research mentorship in LMICs to be included in a research mentorship guide.

## Methods

This study employed a two-round modified Delphi process informed by a related scoping review [11] crowdsourcing open call and an in-person conference [12]. The Delphi process has been widely used to achieve consensus in a structured way. The traditional Delphi process uses iterative survey stages with controlled feedback, statistical group responses including “agreement” levels, as well as solicits the opinions of topic experts [10]. Since the advent of the traditional Delphi process, many researchers have adapted the process to suit their research needs and address potential limitations of the traditional method [13]. While the traditional Delphi method involves anonymous responses, our study was not anonymous as we were interested in measuring the demographics of respondents. Another modification to our study was that we employed a group of panelists to revise the statements instead only one facilitator which is traditional for the Delphi method. Additionally, this modified Delphi process employed a community-based participatory research (CBPR) approach which emphasizes the importance of equity and knowledge sharing in research [14]. We chose to use the Delphi method instead of only in person focus groups at the conference because we wanted to involve as many people as possible. Not everyone on the expert Delphi panel attended the in-person meeting. As such, limiting consensus building to the in person only process would have likely decreased total responses and engagement. Additionally, we wanted to compare conference and non-conference participants to see if being in person had an effect on the level of engagement.

The modified Delphi consensus process included the administration of two online surveys. The surveys were administered two weeks apart from each other in June 2022. We asked participants to indicate their level of agreement with each statement using a 5-point Likert scale (5 = strongly agree, agree, neutral, disagree and 1 = strongly disagree) and participants had the option to provide additional comments on each statement item. We chose to use a 5-point Likert scale due to its simplicity to construct and ease for participants to complete. A 5-point Likert scale produces a simple yet reliable measure of agreement which was necessary for this study [15].

We received a diverse mix of participants across age, gender, role, and work experience. Of the 36 total participants, 61% (22/39) of our participants identified as female while 39% (14/36) identified as male. Additionally, 66% of our participants identified as being from a low-income country or lower-middle income country. Additional information on participants can be found in **Table 1.** Given the focus of the study, for the Delphi we invited persons with interest and experience regarding institutional health research mentorship in LMIC settings.

**Table 1:** Expert participant demographics for the first-round modified Delphi survey.

Table 1 Expert Participant Demographics	
**Characteristics**	**Number (n = 36)**
**Participant’s Sex**	
Male	14 (39%)
Female	22 (61%)
**Highest Degree Completed**	
Bachelor’s Degree	2 (6%)
Master’s Degree	13 (36%)
Doctoral Degree	20 (56%)
Post-Doctoral Degree	1 (3%)
**Age**	
15–30	6 (17%)
30–45	18 (50%)
45–60	11 (31%)
60–75	1 (3%)
75+	0 (0%)
**Country Economic Categorization**	
Low-Income Country	12 (33%)
Lower-Middle Income Country	12 (33%)
Upper-Middle Income Country	7 (19%)
High-Income Country	5 (14%)
**Country**	
Ethiopia	8 (22%)
Nigeria	6 (17%)
Colombia	4 (11%)
United Kingdom	3 (8%)
United States	2 (6%)
China	2 (6%)
Yemen	2 (6%)
Malawi	2 (6%)
Nepal	2 (6%)
Kenya	1 (3%)
Ghana	1 (3%)
Lebanon	1 (3%)
Peru	1 (3%)
Bangladesh	1 (3%)

Consensus panel participants (n = 22) included people identified from the original crowdsourcing open call, steering committee members, and others with expertise. The role of the consensus panel was to put together sections of the consensus document for the research guide and to edit the first draft. Participants included LMIC researchers, mentors, mentees, institutional leaders, and funders. We invited panelists from the open call steering group, finalists and participants on the open call, and members from the collaborating network (TDR Global, AHRI, SESH). There is not enough data from these groups to do a sub analysis of each of these groups, but we asked their identifiers in order to have an idea of the diversity of participants.

Prior to the administration of the modified Delphi surveys, a consensus level of ≥80% was agreed. This is including a response to “agree” and/or “strongly agree” to any statement item on the surveys based on recommendations from the literature. Any statement item from the surveys that achieved 100% agreement were graded as ‘U’ (unanimous); 90%–99% agreement was graded ‘A’; and 80%–89% agreement were graded ‘B’. The consensus panel reviewed the second-round survey grading to determine which items would be included within the final consensus statement for the research mentorship guide.

### Round 1

**Fig 1:**
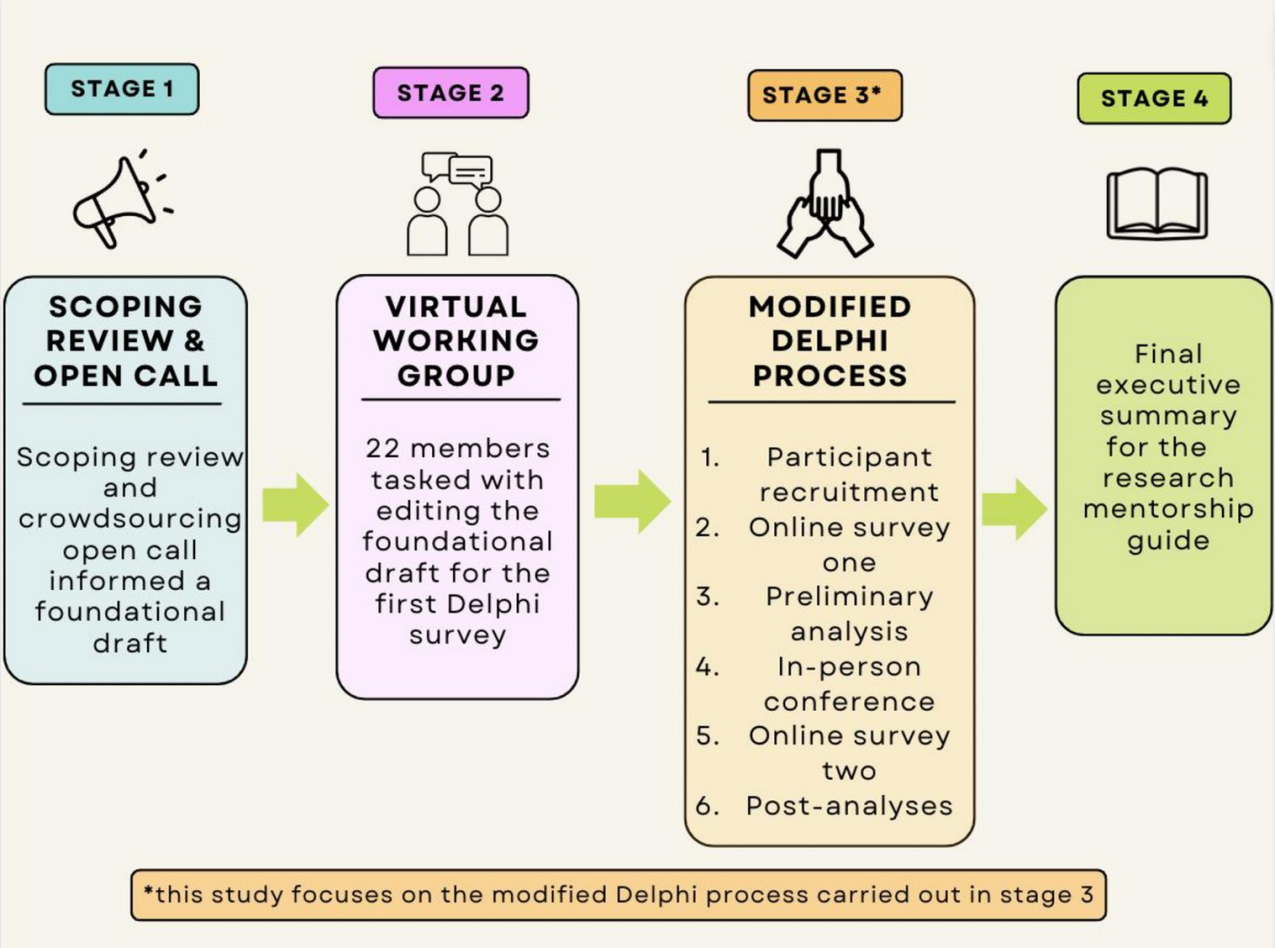
An overview of the four main stages of the institutional research mentorship guide development.

The development of the first-round Delphi survey was based on the statements generated from the open call and scoping review findings by the virtual working group (Fig 1). This was based on existing guidelines on Delphi statement development [16]. The first survey consisted of 39 statement items. The first four items were the participant demographic questions, and the remaining 35 items were the consensus statement items for the research mentorship guide. The facilitator invited participants to the first online survey created with JotForm via email in June 2022.

After participants completed the first-round survey, the core team members compiled the survey results, analyzed consensus level for each statement item and graded them based on the grading scheme described above. We were interested in identifying key themes from the first survey comments. Given that modified Delphi processes are founded on the importance of more than one opinion, we analyzed comments on statement items where two or more participants wrote in similar themes whether it agreed, disagreed, or was neutral with the statement item.

Following the first-round survey, Armauer Hansen Research Institute (AHRI) in partnership with The Special Programme for Research and Training in Tropical Diseases (TDR) hosted an in-person ‘Institutional Research Mentorship Development’ conference in Addis Ababa, Ethiopia from June 23rd to June 24th, 2022. The purpose of the in-person conference was to gather the expert participants to work together on finalizing the research mentorship guide. Having an in-person discussion is useful for consensus building as point of disagreement could be more easily solved in an in person setting. Additionally, we wanted to make sure there was representation from AHRI as they were partners in the study.

### Round 2

During the conference, we created the second-round survey, a modification of the first-round survey based on feedback from round one. The second-round survey was an iteration of the first survey with the comments incorporated from each statement item and from the discussion at the in-person conference in Ethiopia. This survey had 42 items: 1–4 were the participant demographic items, 5–39 were the consensus items and 40–42 were items to correctly acknowledge participants for their contributions to the final practical guide. The conference attendees complete the online survey while in-person in Ethiopia. The research team also sent the second survey via email to the participants from the first survey that were not attending the conference.

We wanted to examine if there were differences between the non-conference participants and the conference participants. We were interested in whether commenting rate (number of participants wrote 2 or more comments) and varied between the two groups.

To measure commenting rate, we looked at how many participants in each group wrote in at least two comments. We took the number of participants who wrote in at least two comments and divided this number by the total participants who responded to come up with a commenting rate.

We also measured the strength of agreement between the two groups. We did this by counting how many statement items participants had marked “strongly agree” over “agree” for the majority of the survey. The majority was calculated by ensuring that over half the number of respondents had responded “strongly agree” per each statement item within each group.

### Informed consent

All participants first completed an informed consent online form as the first part before going ahead to complete the main survey.

### Ethical consideration

The ethics review committees at both the Armauer Hansen Research Institute (AHRI:10–015) and the London School of Hygiene and Tropical Medicine (LSHTM: 27012) granted this study ethics approval.

## Results

There were 61 expert participants recruited for this modified Delphi process however we received survey responses from 36 participants. The full participant demographics can be shown in Table 1 for the first survey.

### Round 1

The first-round survey was built from the foundational draft of the scoping review, open call and from the virtual working groups’ revision. The first survey consisted of 39 statement items. The first four items were the participant demographic questions and the remaining 35 items were the consensus statement items for the research mentorship guide. We emailed out the survey to the 61 participants and we received 36 responses back achieving a response rate of 59%. For round one, consensus was reached (≥ 80% agreement level) on all 35 consensus statement items in the survey. A total of 13 out of the 35 statement items had similar comments written by two or more participants regarding the statement item content.

### Round 2

There were 14 people who joined both days of the conference held in Addis Ababa, Ethiopia. These conference attendees included the crowdsourcing open call finalists, virtual working group members and AHRI researchers. The conference participants met for two days to finalize the institutional research mentorship practical guide and complete the consensus process by revising the first survey through an open discussion.

**Fig 2:**
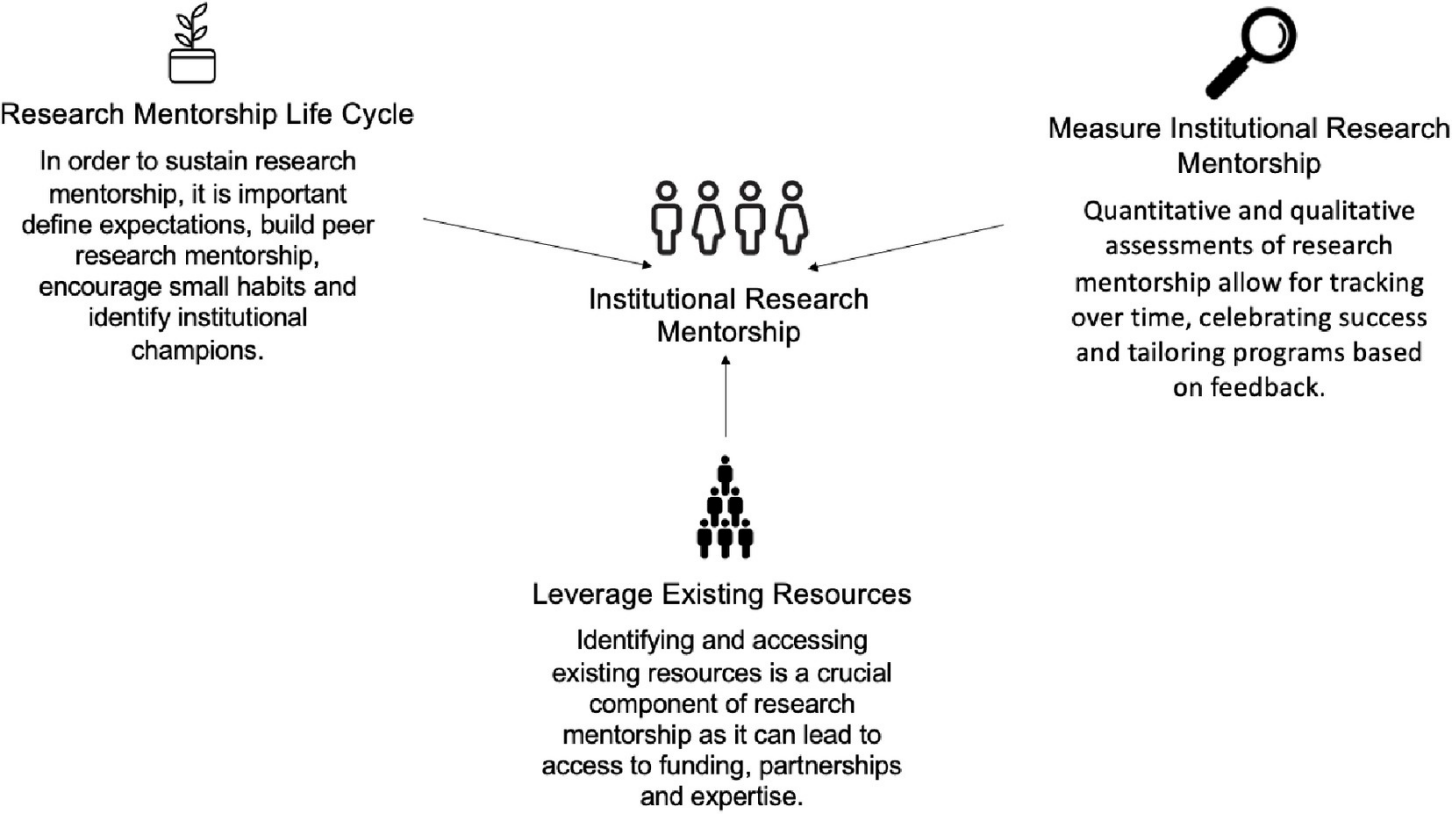
Infographic of the three sections of institutional research mentorship, adapted from the TDR guide.

The second-round survey was an iteration of the first survey with the comments incorporated from each statement item and from the discussion at the in-person conference in Ethiopia. While all the items achieved consensus after Round 1, we wanted to incorporate the feedback received in order to achieve a higher level of consensus. We wanted to ensure that all statement items had been reviewed and revised by our participants. This survey had 42 items: 1–4 were the participant demographic items, 5–39 were the consensus items and 40–42 were items to correctly acknowledge participants for their contributions to the final practical guide. We emailed out the survey to the 61 participants and we received 32 responses back for a response rate of 52%. The participants also reached consensus (**≥** 80% agreement level) on all consensus items in the survey. The development and revision of each statement was a multi-step process and therefore listing the changes at each phase would be beyond the scope of the study. The final consensus statement items and their associated agreement levels is shown in Table 2 below. Fig 2 shows the definition and three main sections of institutional research mentorship that we created through the results of the modified Delphi process [5]. Both Fig 2 and the items in Table 2 were included in the full consensus document for the practical guide (S1 File).

**Table 2:** Final abbreviated consensus statement items with associated degrees of consensus from the second-round modified Delphi survey1.

#	Statement Item	%	Grade
*Definition of Institutional Research Mentorship & Preamble*			
1	We define institutional research mentorship as the institutional-level structures that nurture research capacity within the local institution to improve research effectiveness of the institution.	93	A
2	Infographic (Fig 2)	90	A
3	Preamble: *see appendix*	97	A
*General Guide Statements*			
4	Address intersectional components of mentorship	97	A
5	Research mentorship is a collaborative activity	100	U
6	Acknowledge that local fit is important	97	A
7	Embrace the digital world in sustaining mentorship but remember the power of in-person connections	100	U
8	Consider a holistic approach to career development and mentorship	97	A
9	Utilize local resources for research and research mentorship	100	U
10	Leverage and strengthen the institutional culture of mentorship, inclusivity, and diversity	94	A
11	Cultivating research mentorship is an institutional responsibility	94	A
12	Encourage mentorship from junior colleagues	80	B
*Mentorship Life Cycle*			
13	Identify institutional champions for mentorship	93	A
14	Encourage and reward small habits of routine mentorship	91	A
15	Encourage peer research mentorship	97	A
16	Build common expectations about the mentor-mentee relationship	97	A
*Leveraging Existing Resources*			
17	Mapping and leveraging existing resources is critical	94	A
18	Leverage ongoing research funding	100	U
19	Catalogue existing expertise and identify areas where you do not have local expertise	100	U
20	Leverage ongoing training grants (short and long-term)	100	U
21	Leverage institutions that enhance research mentorship	100	U
22	Twinning brings together relevant mentor-mentee programs/organisations	93	A
23	Ensure research grants support research mentorship	93	A
24	Ensure that research ethical review committees require a capacity building component	90	A
25	Identify ways to embed research mentorship within institutions	100	U
26	Communicate with the broader research community	100	U
*Measuring and Evaluating Institutional Mentorship*			
27	Measuring mentorship is important for continued success	100	U
28	Use feedback to iteratively improve over time	100	U
29	Tailor monitoring and evaluation based on the extent of research mentorship	93	A
30	Quantitative measurement of research mentorship	97	A
31	Qualitative measurement of research mentorship	97	A
32	Celebrate success within research teams	100	U
33	Tracking and system of documentation of mentorship activities at the different levels of the program process	100	U
34	Develop tools to measure the institutionalization of research mentorship	100	U
35	Recommended open access resources: *see appendix*	94	A

^1^ Statements with 100% agreement were graded as ‘U’ (unanimous); 90%–99% agreement was graded ‘A’; and 80%–89% agreement were graded ‘B’.

### Post-modified Delphi process analyses

We sent the second-round survey to 14 conference participants and 47 non-conference participants. Of the 14 conference participants, 11 responded to the survey for a response rate of 79%. Of the non-conference participants, 21 responded to the survey for a response rate of 45%. For measuring engagement, in the conference group all 11 participants who responded had written in comments for at least 2 statement items. They had a commenting rate of 100%. For the 21 non-conference participants who responded, 8 wrote in comments for at least 2 statement items with an engagement level of 38%.

## Discussion

We developed a consensus document (Appendix 1) to enhance research mentorship in LMIC institutions. In both rounds of the modified Delphi surveys all consensus items reached the predetermined consensus level of ≥ 80%. Through the Delphi study, we were able to achieve our key objective which was to develop a consensus document on institutionalizing research mentorship. The HERMES guide was published in October of 2022. We were also able to meet our secondary objective which was to compare engagement levels of conference and non-conference participants to see if being in person had an effect on engagement. This study expands the literature by using crowdsourcing in a consensus process.

In our analyses we found an increased survey response rate and engagement rate for the conference participants compared to the non-conference participants. This is consistent with the findings of other studies that measured engagement rates of online conferences [17–19]. Both groups were informed that they would be able to get access to the final guide and receive credit for their contributions if desired. The response rate may have been higher for the in-person group because they were given dedicated time to complete the survey. Additionally, by virtue of this group being physically at the conference with the common goal to produce a guide from which they would all benefit, these participants may have been more invested in seeing the final product come to fruition. We also found that the in-person conference did not artificially increase the strength of agreement by means of collective conversation or other group mentality, but rather both the in-person and at-home participants were equally strong in their agreement levels. This stands in contrast to other reported observations, where the sole use of conferences dedicated to consensus can take longer to execute and can be more susceptible to group conformity [20]. However, we are recommending future Delphi studies to include both virtual and in-person aspects. As previously mentioned, an in-person aspect is important for connection and consensus building while a virtual aspect ensures diversity and inclusion of all participants.

This modified Delphi process helped show key aspects of institutional research mentorship that researchers in LMICs found important. These key aspects included the three sections that informed the definition of institutional research mentorship: the research mentorship life cycle, leveraging of existing resources and the measurement of institutional research mentorship (Fig 2). Based on the preliminary analysis of the key themes from the first-round survey (Table 2) it was evident that the participants valued the inclusion of equity and fair, dynamic processes within the final practical research mentorship guide. There was also an emphasis placed on considering current cultural practices within institutions as the institutions themselves begin to foster research mentorship. Additionally, the participants acknowledged that institutions should consider appointing a mentorship leader or dedicating an office to mentorship in order to truly embed mentorship into the institution and sustain the practice.

Our study has several limitations. First, there is no standardized way to measure the reliability of a modified Delphi process [21]. However, we used established methods [22] and complemented them with participatory crowdsourcing methods. Second, not all participants attended the in-person conference in Ethiopia. To mitigate this problem, we organized a hybrid meeting session during the in-person conference where working group members not attending could join remotely. Third, the framing of the open call focused on strategies to enhance research mentorship and not barriers to research mentorship. As a result, less can be stated about barriers to institutional research mentorship. Specific to our application of this modified Delphi process was the potential limitation of the survey being too long for participants to remain engaged or to be willing to complete both surveys. This limitation is often referred to as survey fatigue [23]. However, our results were encouraging in that the response rate only decreased by 7% between the two surveys with over 50% of participants responding in both.

This study has implications for public health policy and research. From a policy perspective, the use of remote data collection capabilities reduced the cost and time-to-consensus of the decision-making process [24]. Including both an in-person and virtual option was key to the success of our consensus process. Additionally, this consensus process helped in the development of the HERMES practical guide which contains methods for enhancing institutional research mentorship. The guide has been disseminated across the various TDR Global partner organizations. The HERMES guide provides general ideas for enhancing research mentorship in LMICs, however, there are many areas that could be further explored such as gender and age. We suggest that further research be conducted into understanding and quantifying the value of having multiple modalities in the consensus building process. A gap currently exists in the literature regarding clear guidelines for qualitative data analyses of modified Delphi processes [25]. Assessment of the Delphi process could help to iteratively improve the method.

## Conclusion

With a 2 round modifies Delphi process, we achieved consensus on strategies to improve research mentorship among LMIC organizations. The results from this study have directly informed a World Health Organization practical guide that is now being disseminated [5]. However, there remains unanswered questions and areas that require further research and policy attention.

## Supporting information

S1 Checklist STROBE statement—checklist of items that should be included in reports of observational studies. (DOCX)

S1 File Consensus document (Full version). (DOCX)

S2 File Delphi survey questionnaire for round 1: https://form.jotform.com/221463008762351. (DOCX)

S3 File Delphi survey questionnaire for round 2: https://eu.jotform.com/221691037864359. (DOCX)

## References

[1] Homer H. The odyssey: Xist Publishing; 2015.

[2] Gagliardi AR, Webster F, Perrier L, Bell M, Straus S. Exploring mentorship as a strategy to build capacity for knowledge translation research and practice: a scoping systematic review. Implementation Science. 2014;9(1):1–10. 25252966 10.1186/s13012-014-0122-z PMC4182766

[3] Hollingsworth MA, Fassinger RE. The role of faculty mentors in the research training of counseling psychology doctoral students. Journal of Counseling Psychology. 2002;49(3):324.

[4] Davis SN, Garner PW, Jones RM, Mahatmya D. The role of perceived support and local culture in undergraduate research mentoring by underrepresented minority faculty members: findings from a multi-institutional research collaboration. Mentoring & Tutoring: Partnership in Learning. 2020;28(2):176–88.

[5] Ethiopia S, Organization WH. Health research mentorship in low-and middle-income countries (HERMES): a TDR global practical guide to spur mentorship institutionalization: World Health Organization; 2022.

[6] Hansoti B, Kalbarczyk A, Hosseinipour MC, Prabhakaran D, Tucker JD, Nachega J, et al. Global health mentoring toolkits: a scoping review relevant for low-and middle-income country institutions. The American Journal of Tropical Medicine and Hygiene. 2019;100(1 Suppl):48. doi: 10.4269/ajtmh.18-0563 30430981 PMC6329353

[7] Schwerdtle P, Morphet J, Hall H. A scoping review of mentorship of health personnel to improve the quality of health care in low and middle-income countries. Globalization and Health. 2017;13:1–8. 28974233 10.1186/s12992-017-0301-1 PMC5627414

[8] Lescano AG, Cohen CR, Raj T, Rispel L, Garcia PJ, Zunt JR, et al. Strengthening mentoring in low-and middle-income countries to advance global health research: an overview. The American journal of tropical medicine and hygiene. 2019;100(1 Suppl):3. 10.4269/ajtmh.18-0556 PMC6329352 30430982

[9] Oppong E, Bao H, Tang W, Mejia MIE, Glozah F, Asanga N, et al. A global crowdsourcing open call to improve research mentorship in low-and middle-income countries: a mixed methods analysis. Am J Trop Med Hyg. 2021;106(1):250–6. doi: 10.4269/ajtmh.21-0607 34662869 PMC8733547

[10] Mubarak N, Hatah E, Aris MAM, Shafie AA, Zin CS. Consensus among healthcare stakeholders on a collaborative medication therapy management model for chronic diseases in Malaysia; a Delphi study. PloS one. 2019;14(5):e0216563. doi: 10.1371/journal.pone.0216563 31075110 PMC6510413

[11] Yoseph Abraha KMEEKMKZBAAJT. Research Mentorship in Low and Middle-Income Countries: A Scoping Review 2022 [updated 2022-07-29. Available from: https://osf.io/jqa9z/.

[12] Kpokiri EE, McDonald K, Gebreyohannes Y, Osorio L, Nath TC, Talavera-Urdanivia VA, et al. Research Mentorship in Low and Middle-Income Countries: A Global Qualitative Evidence Synthesis of Data from a Crowdsourcing Open Call and Scoping Review. medRxiv. 2022. 10.1136/bmjgh-2022-011166 PMC10773352 38184299

[13] Hasson F, Keeney S, McKenna H. Research guidelines for the Delphi survey technique. Journal of advanced nursing. 2000;32(4):1008–15. 11095242

[14] Suto MJ, Lapsley S, Balram A, Barnes SJ, Hou S, Ragazan DC, et al. Integrating Delphi consensus consultation and community-based participatory research. Engaged Scholar Journal: Community-Engaged Research, Teaching, and Learning. 2019;5(1):21–35.

[15] Bertram D. Likert scales. Retrieved November. 2007;2(10):1–10.

[16] Hsu C-C, Sandford BA. The Delphi technique: making sense of consensus. Practical assessment, research, and evaluation. 2007;12(1):10.

[17] Raby CL, Madden JR. Moving academic conferences online: Aids and barriers to delegate participation. Ecology and Evolution. 2021;11(8):3646–55. doi: 10.1002/ece3.7376 33898017 PMC8057330

[18] Wood L, Bjarnason GA, Black PC, Cagiannos I, Heng DYC, Kapoor A, et al. Using the Delphi Technique to Improve Clinical Outcomes Through the Development of Quality Indicators in Renal Cell Carcinoma. Journal of Oncology Practice. 2013;9(5):e262–e7. doi: 10.1200/JOP.2012.000870 23943895

[19] Bleijlevens MH, Wagner LM, Capezuti E, Hamers JP, Workgroup IPR. Physical restraints: consensus of a research definition using a modified delphi technique. Journal of the American Geriatrics Society. 2016;64(11):2307–10. doi: 10.1111/jgs.14435 27640335

[20] Chen D-S, Deng C-Y. Interaction between citizens and experts in public deliberation: A case study of consensus conferences in Taiwan. East Asian Science, Technology and Society: An International Journal. 2007;1(1):77–97.

[21] Sackman H. Summary Evaluation of Delphi. Policy Analysis. 1975;1(4):693–718.

[22] Lange T, Kopkow C, Lützner J, Günther K-P, Gravius S, Scharf H-P, et al. Comparison of different rating scales for the use in Delphi studies: different scales lead to different consensus and show different test-retest reliability. BMC medical research methodology. 2020;20(1):1–11. doi: 10.1186/s12874-020-0912-8 32041541 PMC7011537

[23] Porter SR, Whitcomb ME, Weitzer WH. Multiple surveys of students and survey fatigue. New directions for institutional research. 2004;2004(121):63–73.

[24] Hensen B, Mackworth-Young CRS, Simwinga M, Abdelmagid N, Banda J, Mavodza C, et al. Remote data collection for public health research in a COVID-19 era: ethical implications, challenges and opportunities. Health Policy and Planning. 2021;36(3):360–8. doi: 10.1093/heapol/czaa158 33881138 PMC7928874

[25] Brady SR. Utilizing and Adapting the Delphi Method for Use in Qualitative Research. International Journal of Qualitative Methods. 2015;14(5):1609406915621381.

