# Supplementary material for: Development of a research mentorship guide and consensus statement for low- and middle-income countries: Results of a modified Delphi process
Source: PLoS One. 2023 Oct 25;18(10):e0291816. doi: 10.1371/journal.pone.0291816 (PMC10599585; doi:10.1371/journal.pone.0291816)
Supplement: S1 File — (DOCX) [file pone.0291816.s002.docx]

Consensus Document (Full Version)

| **Number** | **Statement Item** | **%** | **Grade** |
| --- | --- | --- | --- |
| *Definition of Institutional Research Mentorship & Preamble* | | | |
| 1. | We define institutional research mentorship as the institutional-level (individuals, universities, professional associations, and research institutes) structures that nurture research capacity within the local institution to improve research effectiveness of the institution. | 93 | A |
| 2. | Infographic (Figure 2) | 90 | A |
| 3. | Preamble: Research mentorship can instill research culture, improve scientific and grant writing, promote career development, and mould generations of researchers. Many research mentorship frameworks and tools that exist are designed for use in high-income settings. However, these frameworks and tools are often difficult to implement and sustain in LMICs because of structural differences, cultural factors, infrastructural issues, and capacity limitations. This calls for practical guidance to build and further develop institutional research mentorship in LMICs. In this guide, institutional research mentorship is defined as the institutional-level (individuals, universities, professional association, and research institutes) structures that nurture research capacity within the local institution to improve research effectiveness of the institution. This document developed through a crowdsourcing contest, an online Delphi method and a scoping review of evidence can be used to embed and deliver a more productive and fruitful institutional research mentorship guide for LMICs. This practical guide does not focus on how to be a good mentee or mentor as there are numerous other resources on these topics. The intended audiences for this guide are institutional leaders, government agencies, funders, and others interested in building institutional research mentorship in LMICs or other resource-constrained settings. | 97 | A |
| *General Guide Statements* | | | |
| 4. | Address intersectional components of mentorship: Research mentorship has a specific intersectional context that often disadvantages specific people in systematic ways. In this guide “intersectional” includes race, class, gender and ability/disability levels. Empowering disadvantaged groups and providing structured, systematic, inclusive and equitable ways to enhance research mentorship is vital. | 97 | A |
| 5. | Research mentorship is a collaborative activity: Institutional research mentorship requires support from groups at several levels (research team, university, government, professional associations, institutions, funders) in order to be sustainable and thrive over time. | 100 | U |
| 6. | Local fit is important: There is no monolithic way to start or sustain research mentorship in LMICs. There is substantial heterogeneity in how research mentorship can be cultivated, and the practical guide may require adaptation including the use of local languages and alterations according to specific local needs and contexts. | 97 | A |
| 7 | Embrace the digital world in sustaining mentorship but remember the power of in-person connections: The digital world creates many opportunities for enhancing research mentorship, including intra-country and inter-regional connections. At the same time, there is inherent value to in-person meetings and connection to advance research mentorship outcomes and performance. | 100 | U |
| 8. | Consider a holistic approach to career development and mentorship: Many exceptional trainees will face competing priorities from home and social lives including family life. These personal aspects of mentorship should be acknowledged as part of a holistic approach to personal growth and leadership development. A genuine interest in overall well-being, beyond simply professional development, should be nurtured. | 97 | A |
| 9. | Local resources for research and research mentorship: In order to introduce and sustain a culture of research, identifying relevant local resources (e.g., senior research staff, junior investigators, research funding, institutional leadership) is important to ensure continued and long-lasting success. | 100 | U |
| 10. | Leverage and strengthen the institutional culture of mentorship, inclusivity, and diversity: by embedding mentorship into the DNA of institutions, it becomes more sustainable and not dependent on an individual champion. We would encourage institutions to consider mentorship in promotion policies. | 94 | A |
| 11. | Cultivating research mentorship is an institutional responsibility: All trainees need mentorship, support and guidance. Although many programs infer that only exceptional trainees need mentorship, we believe that everyone can benefit from mentorship. | 94 | A |
| 12. | Mentorship from junior colleagues: Creating a reverse system where junior colleagues have opportunities to provide mentorship for senior colleagues in order to dismantle power structures, identify systematic bias and increase equity. | 80 | B |
| *Mentorship Life Cycle* | | | |
| 13. | Identify institutional champions for mentorship: Mapping key stakeholders working with institutions at local, national, regional levels and beyond for improved research practices and outcomes. Champions include researchers, institutional leaders, groups, and students for institutional research mentorship. | 93 | A |
| 14. | Encouraging and rewarding small habits of routine mentorship: Develop and promote existing pay-it-forward initiatives through tailored capacity building and the recognition of best practices. | 91 | A |
| 15. | Peer research mentorship should be encouraged: Peer mentorship refers to someone providing practical advice to someone at a similar career stage. Peer mentorship and introducing junior scientists, including Ph.D. students, into mentoring early on will create a culture of mentorship that is sustainable over time. | 97 | A |
| 16. | Build common expectations about the mentor-mentee relationship: Building common expectations about the mentor-mentee relationship, the frequency of meetings, accountability mechanisms, conflict management and resolution. This may include a written individualised development plan. | 97 | A |
| *Leveraging Existing Resources* | | | |
| 17. | Mapping and leveraging existing resources is critical: Institutional research mentorship can be cultivated and nurtured in low-resource settings. Identifying existing resources to support research mentorship is an important strategy that is useful in a wide range of LMIC settings. | 94 | A |
| 18. | Leverage ongoing research funding: Ongoing research grants can provide a strong foundation for cultivating research mentorship by including graduate, postgraduate students, and other trainees. Direct costs for research can be used to employ research assistants, provide mentored research opportunities, and cultivate mentor-mentee relationships. This provides a way to embed mentorship in research routine activities. | 100 | U |
| 19. | Catalogue existing expertise and identify areas where you do not have local expertise: This information can be used as a resource for mentees to approach potential local mentors. Keeping this catalogue of institutional expertise and potential mentors as well as national and international partners should be accessible on-line and should be maintained as a standard practice. Potential mentorship resources and mentors outside the institution should also be documented. | 100 | U |
| 20. | Leverage ongoing training grants (short and long-term): National, regional, and global training programs can provide practical strategies to enhance institutional research mentorship and the tools can be widely adopted. For example, TDR and Fogarty both support LMIC researchers undertaking mentored research. | 100 | U |
| 21. | Leverage institutions that enhance research mentorship: Consider the professional associations, universities, government organisations, and other institutions such as relevant government bodies (ministries of health, science, innovation and technology) that promote research mentorship. This can help to leverage existing structures, competencies, and research infrastructure. Identifying these key champions can help create collaborative networks that support institutional mentorship. | 100 | U |
| 22. | Twinning brings together relevant mentor-mentee programs/organisations: Consider the professional associations, universities, government organisations, and other institutions such as relevant government bodies (ministries of health, science, innovation and technology) that promote research mentorship. This can help to leverage existing structures, competencies, and research infrastructure. Identifying these key champions can help create collaborative networks that support institutional mentorship. | 93 | A |
| 23. | Ensure research grants support research mentorship: Institutions should require pre-award budget details to explicitly include key direct costs associated with research mentorship (e.g., graduate research assistants, salary support for mentors). | 93 | A |
| 24. | Ensure that research ethical review committees require a capacity building component: Research ethical review committees should require consideration of local capacity building and mentorship. This could include formal technology transfer, capacity building workshops, pairing of scholars, and other mechanisms. | 90 | A |
| 25. | Identify ways to embed research mentorship within institutions: In addition to financial support from training grants and local institutions, alternative financing strategies (e.g., crowdfunding) can help to sustain research mentorship over time. In addition, having elective courses to support mentorship should be considered. | 100 | U |
| 26. | Communication with the broader research community: Sustaining research mentorship over time requires identifying and cultivating talent within organisations, promotion and recognition of people with mentorship experience, and matching of mentor-mentee based on relevant criteria such as expertise, and research interests. | 100 | U |
| *Measuring and Evaluating Institutional Mentorship* | | | |
| 27. | Measuring mentorship is important for continued success: Measurement and evaluation of research mentorship is essential for demonstrating success, rewarding exemplars and effective mentorship, identifying areas for improvement, and sustaining institutional mentorship over time. Feedback should be for both mentors and mentees. | 100 | U |
| 28. | Using feedback to iteratively improve over time : Establishing effective, structured feedback is crucial to improve mentorship experiences and outcomes; reduce intentional bias; and protect both mentors and mentees. In line with this, it is crucial to establish mechanisms to mitigate negative mentorship experiences within the established framework of mentor-mentee relationships. | 100 | U |
| 29. | Tailoring monitoring and evaluation based on the extent of research mentorship: Adapting measurement metrics based on how far ongoing mentorship programmes have advanced. | 93 | A |
| 30. | Quantitative measurement of research mentorship: Quantitative metrics to understand the effectiveness of research mentorship include the number of trainees, the number of trainees retained in research over time, the number of trainees who secure independent research positions, the number of trainees who secure independent research funding, the number of research mentors, the number of research mentors. Additional outcomes to consider include confidence and independency, perceived skills and capacity, research identity, research ethics, integrity, relationship quality, attitudinal, and psychological well-being. | 97 | A |
| 31. | Qualitative measurement of research mentorship: Qualitative analysis of research mentorship is important to understand gender dynamics and disparity, racial, and intersectional issues that serve as structural and socio-economic barriers and facilitators to institutional mentorship in LMICs. This could include semi-structured interviews, in-depth interviews, focus group discussions, or crowdsourcing open calls. | 97 | A |
| 32. | Celebrate success within research teams: Recognize that research is a team effort, provide incentives, awards, and formal recognition of the units, teams, and structures that nurture research mentorship. For example, give awards not to an individual mentor or mentee, but to a research group, department, or unit. | 100 | U |
| 33. | Tracking and system of documentation of mentorship activities at the different levels of the program process: Inputs, activities, outputs and impacts (individual/mentor/mentee, institutional level). | 100 | U |
| 34. | Develop tools to measure the institutionalisation of research mentorship: Further research is needed to develop tools to measure research mentorship within research and academic institutions through formal curricula, assigning responsibilities to existing units, or creating new units. | 100 | U |
| 35. | Recommended open access resources | 94 | A |
