# Supplementary figures and images for: Development of a research mentorship guide and consensus statement for low- and middle-income countries: Results of a modified Delphi process
Source: PLoS One. 2023 Oct 25;18(10):e0291816. doi: 10.1371/journal.pone.0291816 (PMC10599585; doi:10.1371/journal.pone.0291816)

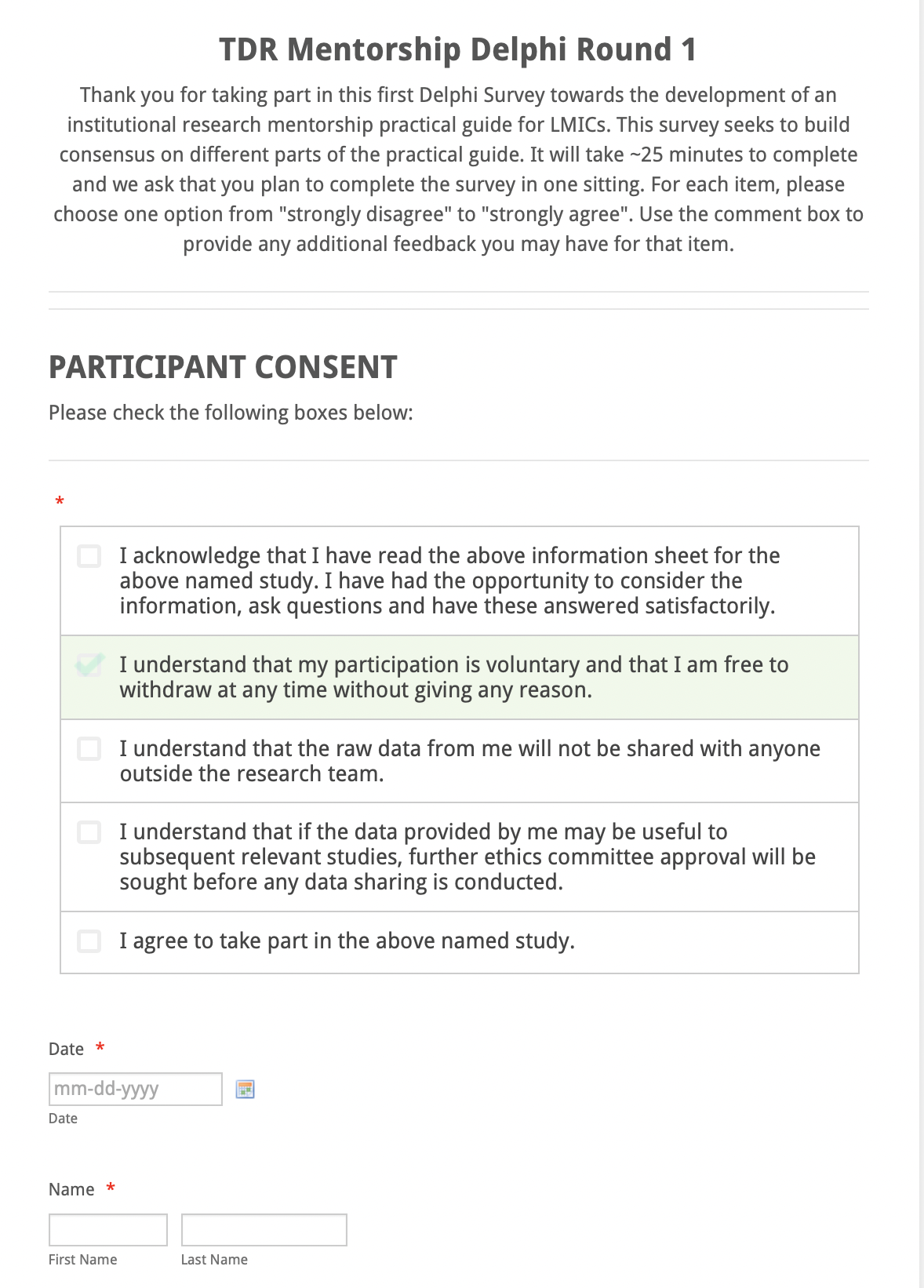

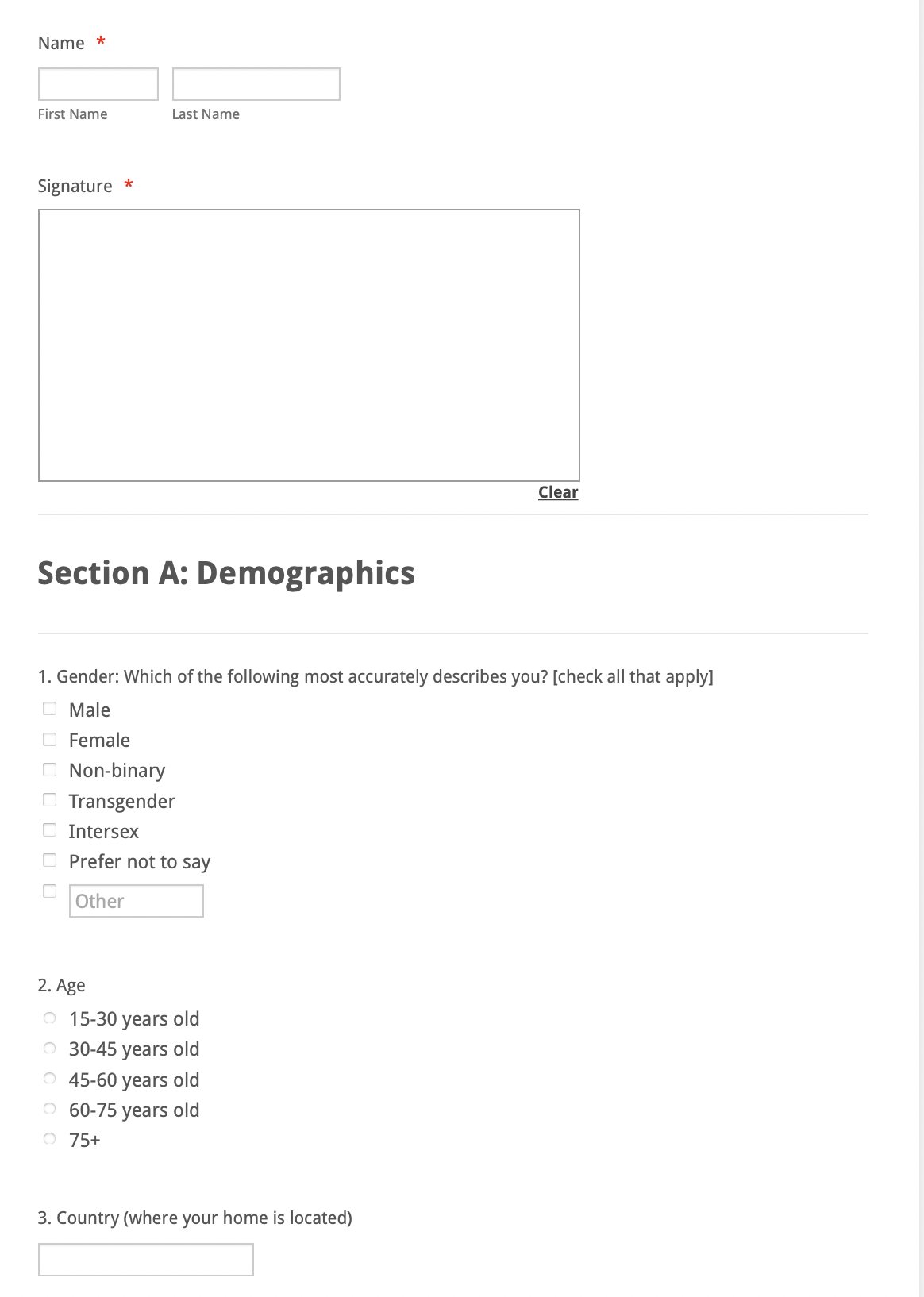

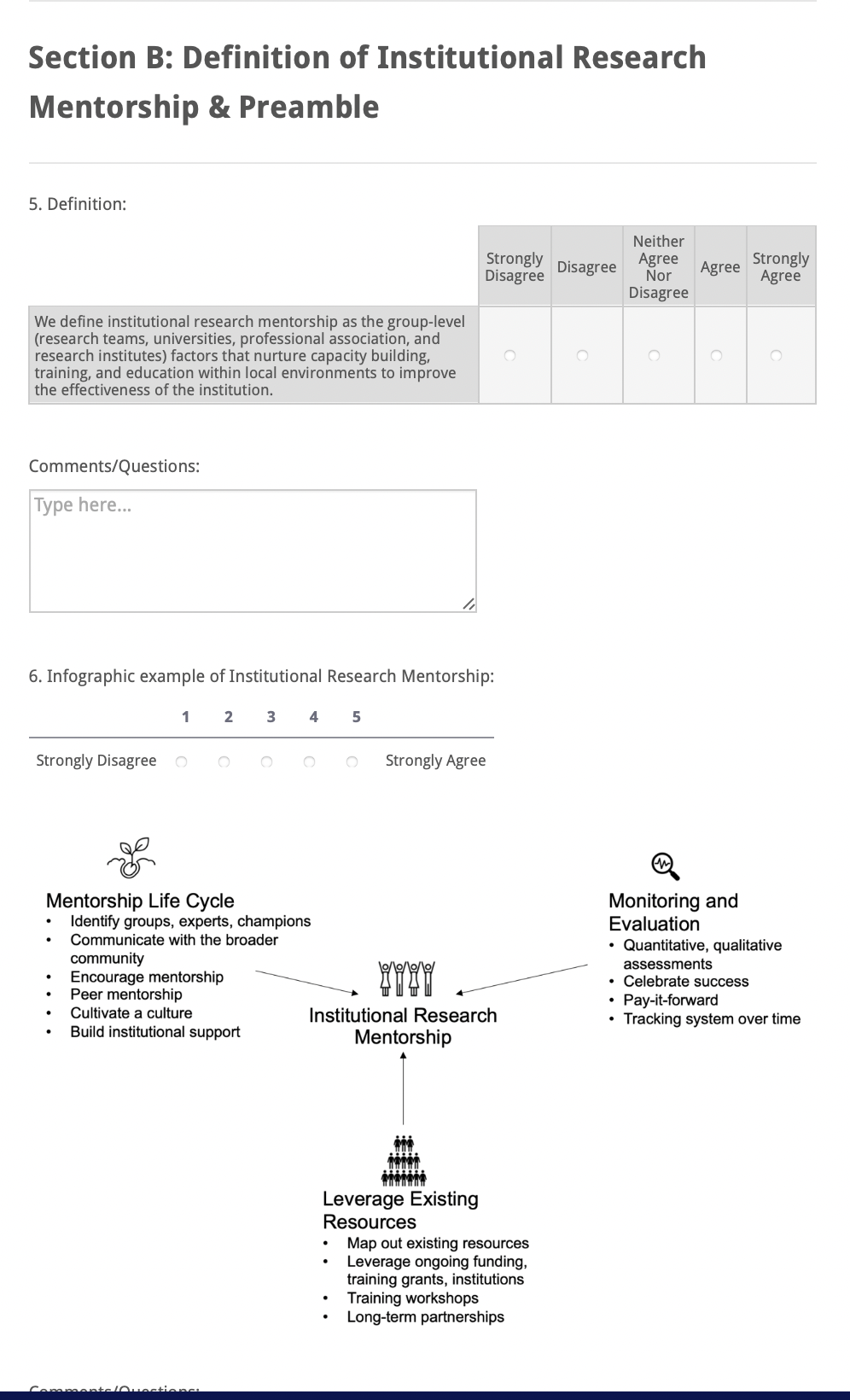

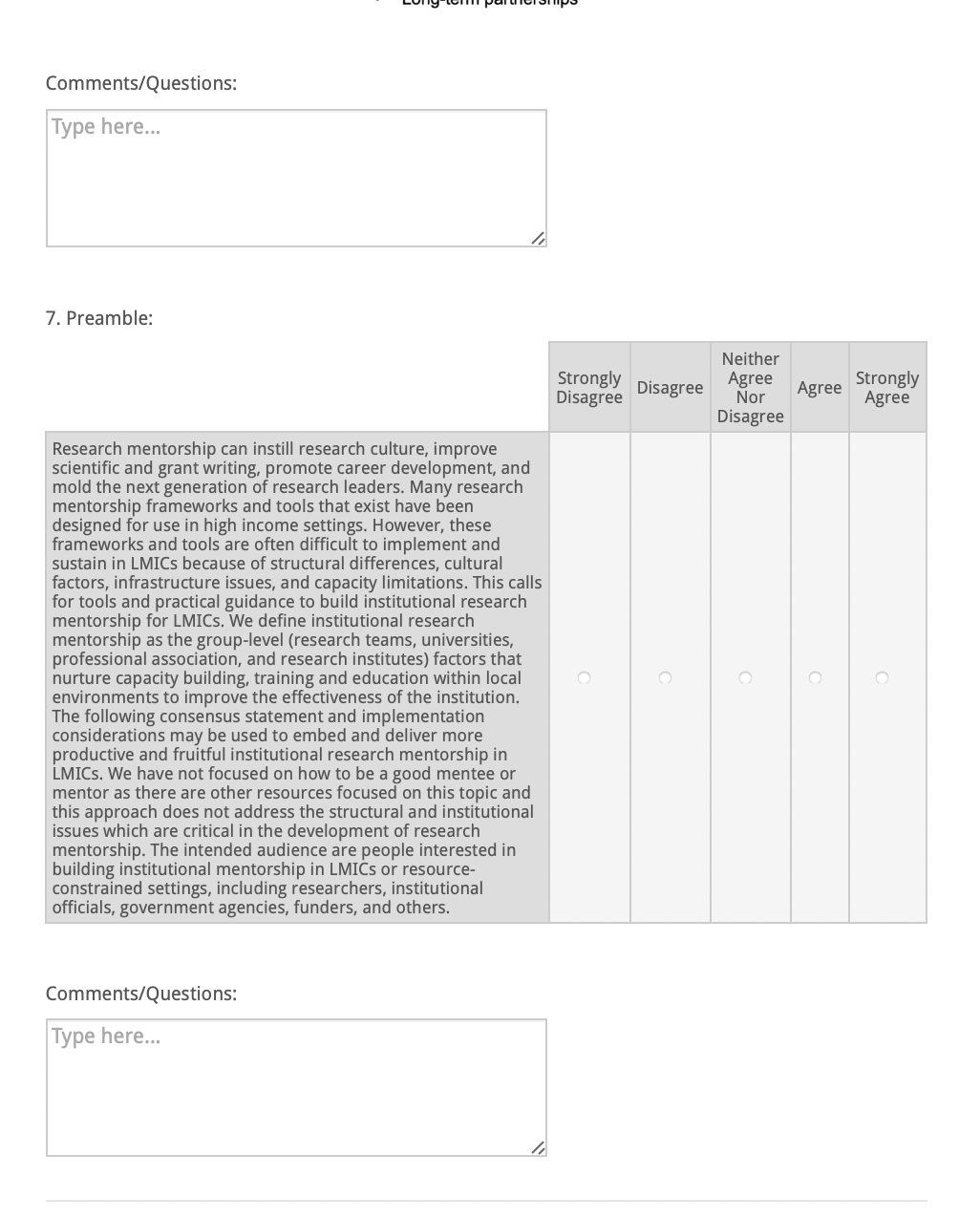

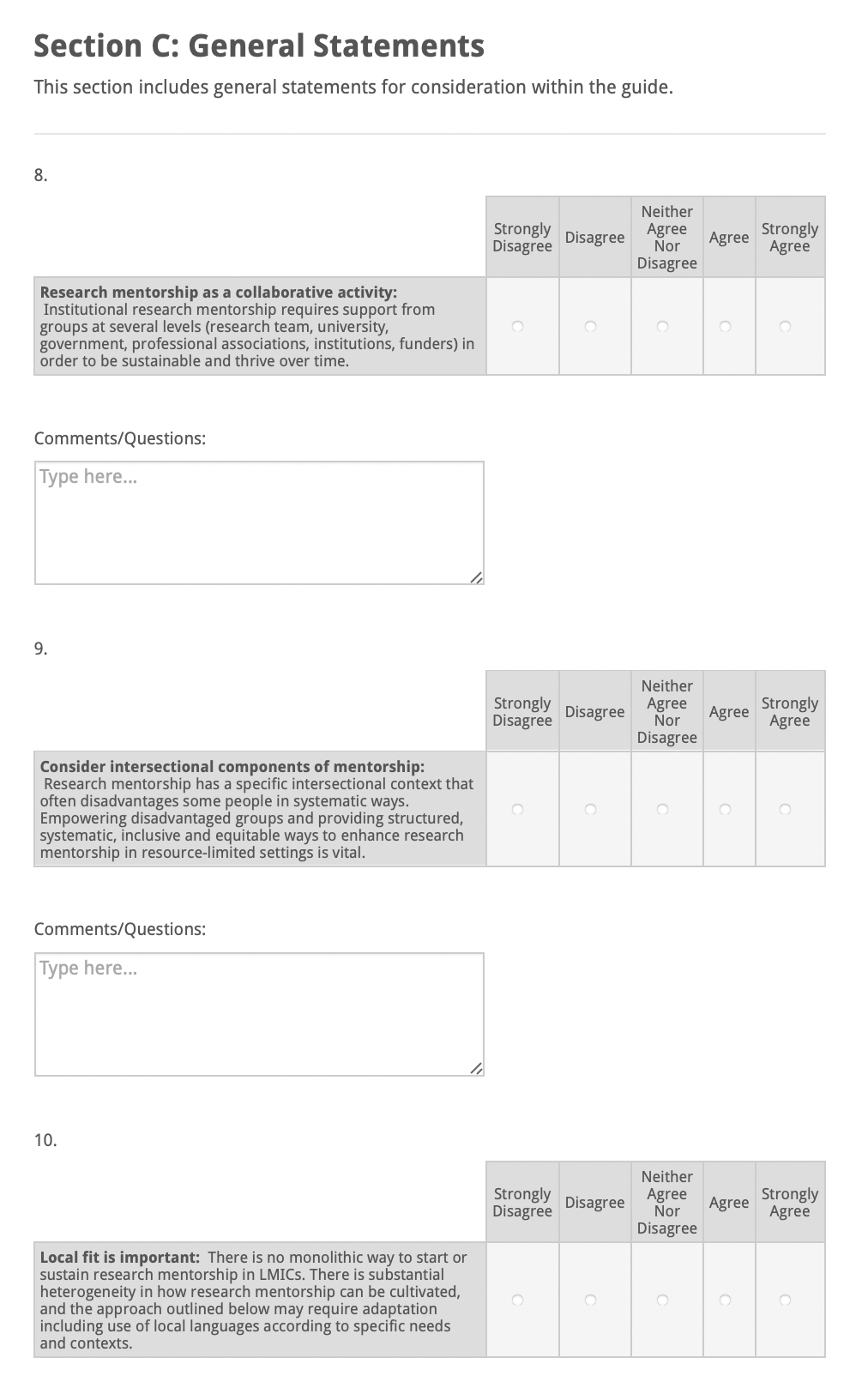

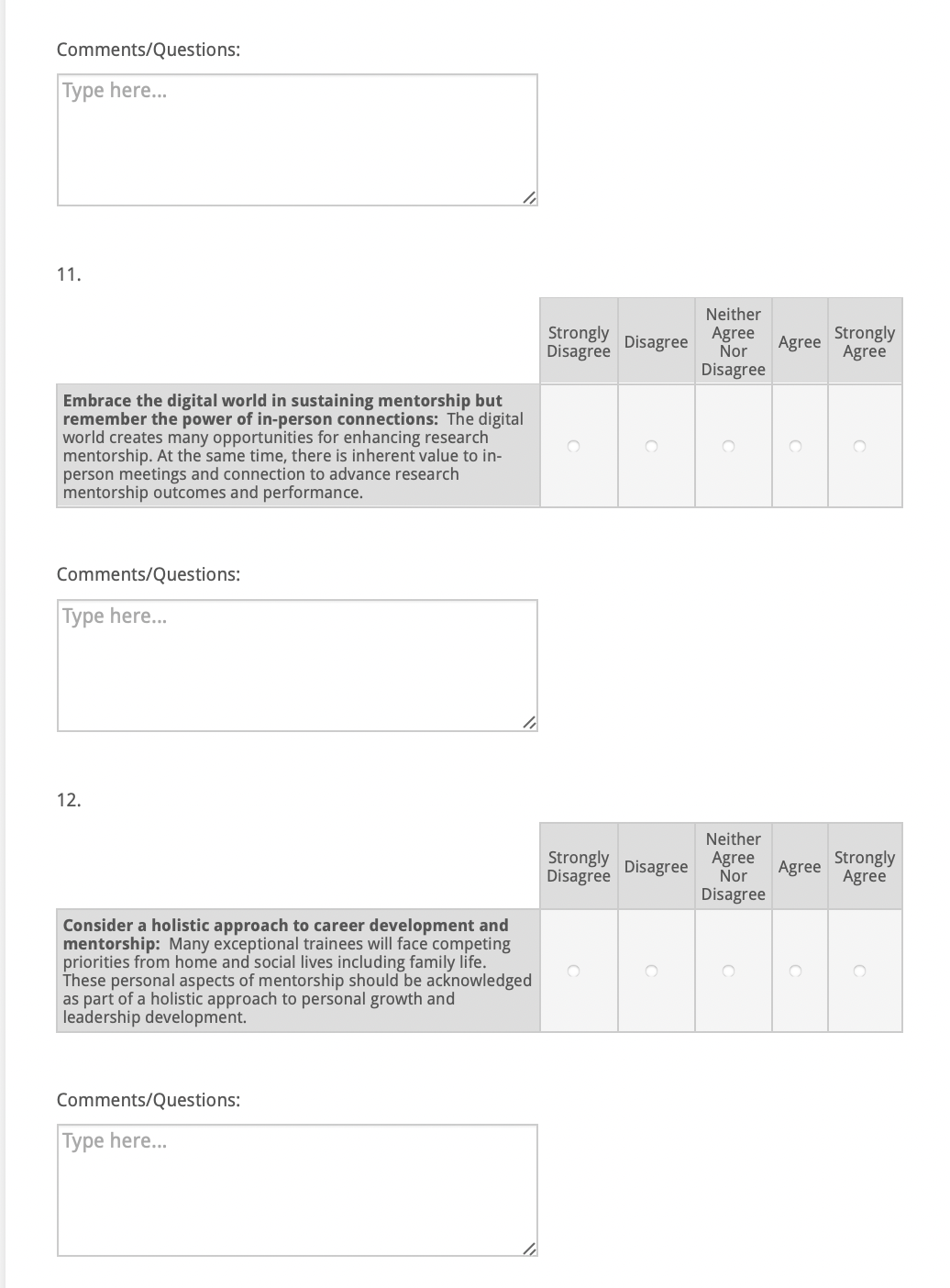

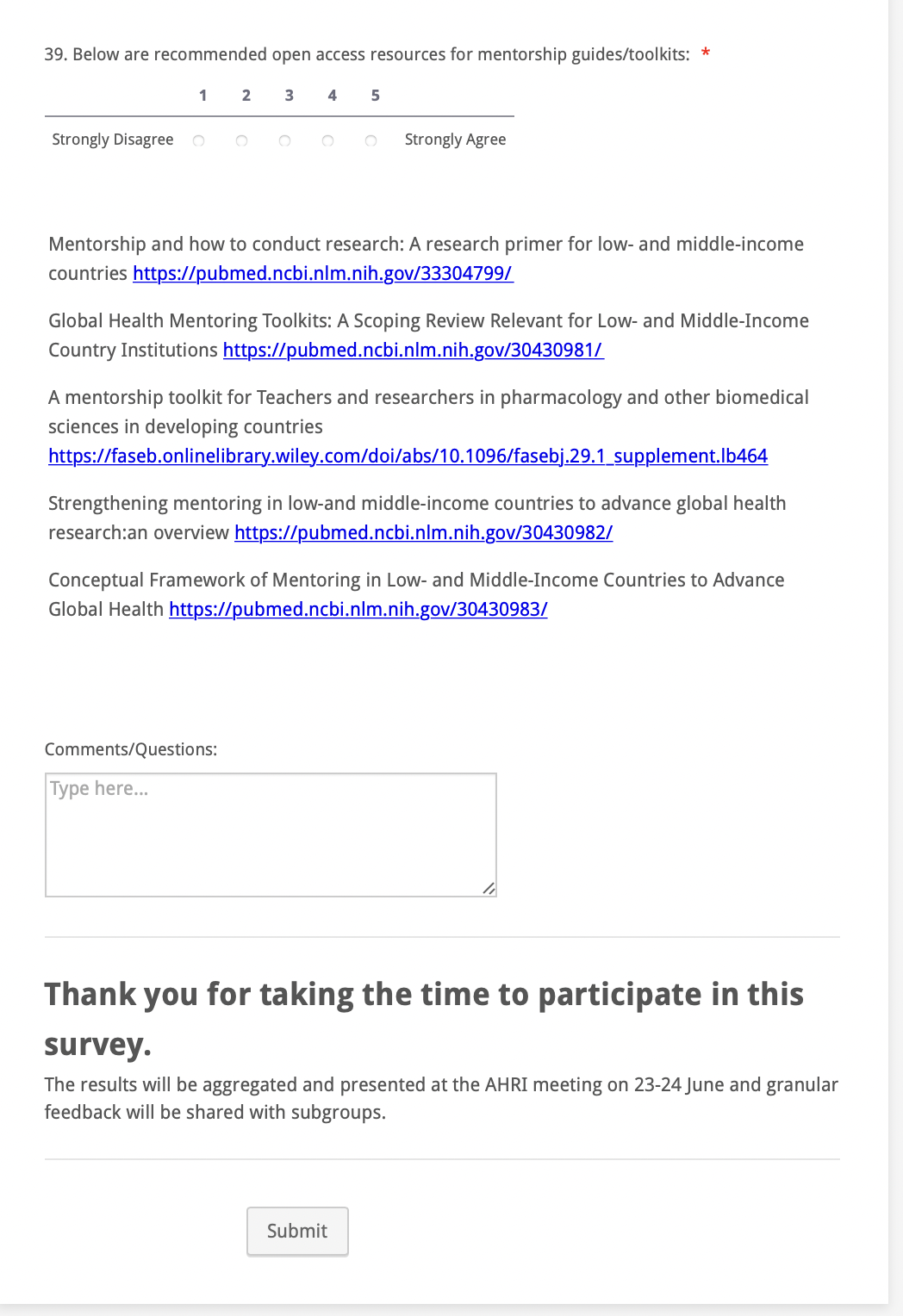

Supplement: S2 File — (DOCX) [file pone.0291816.s003.docx]

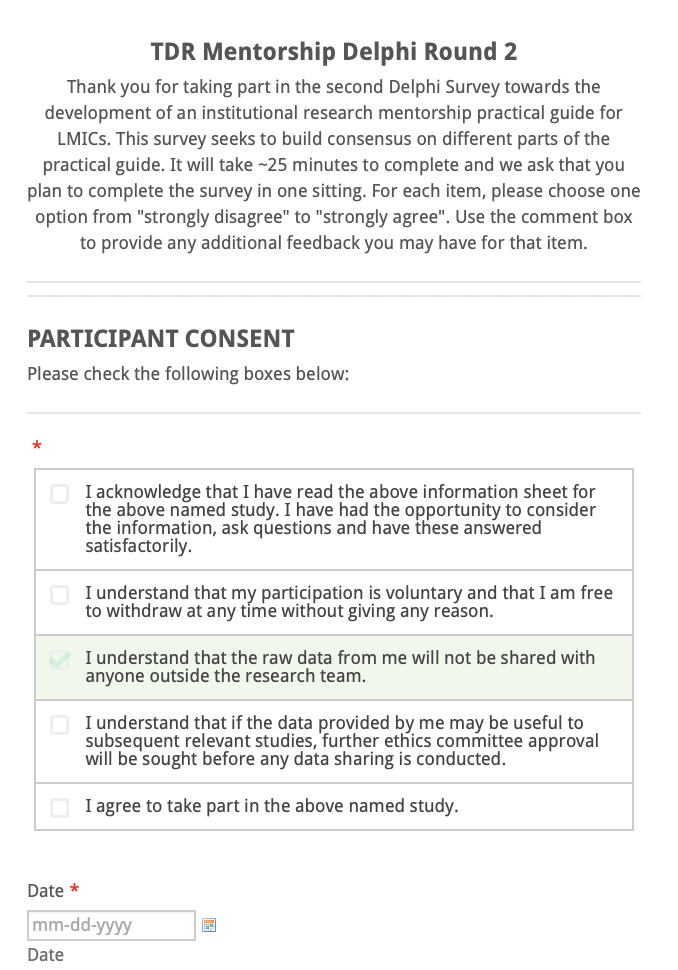

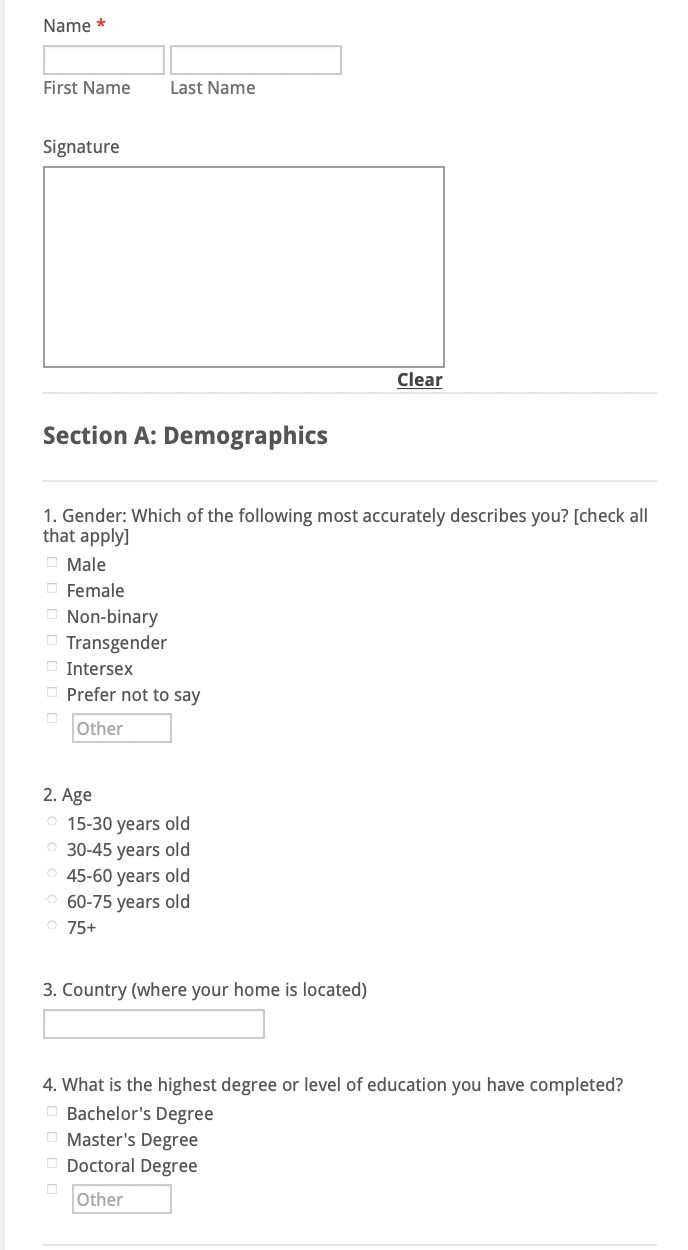

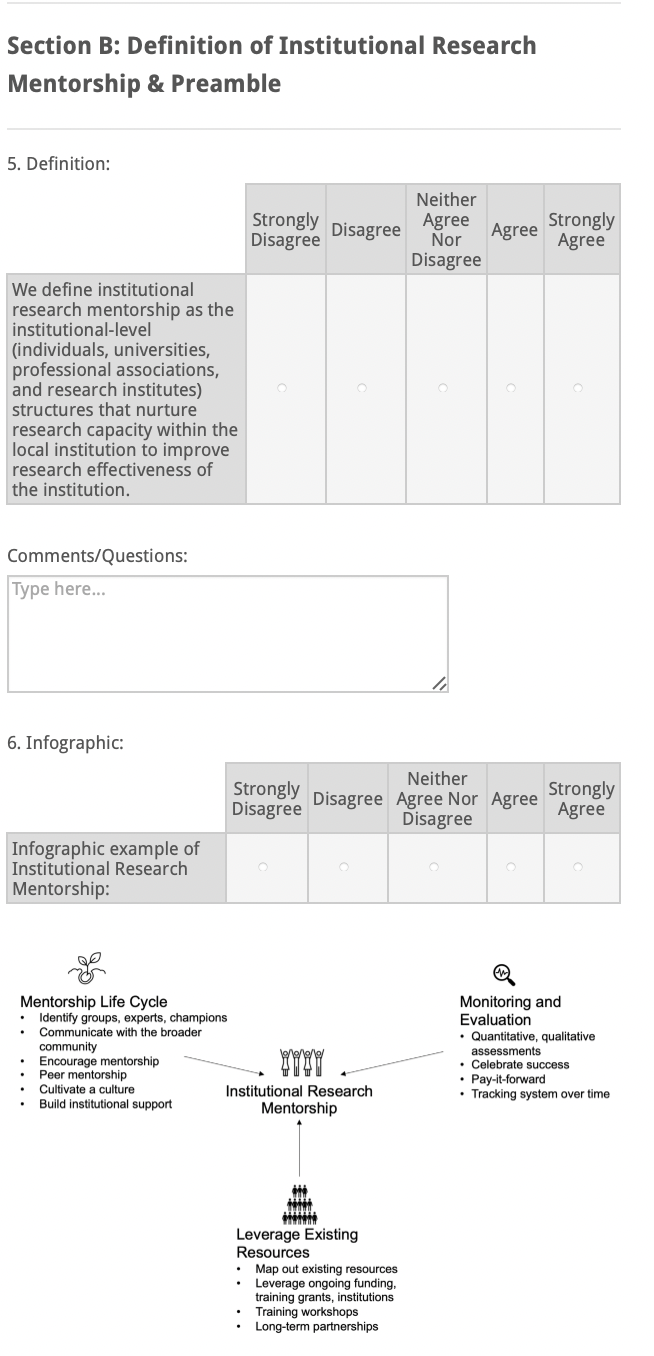

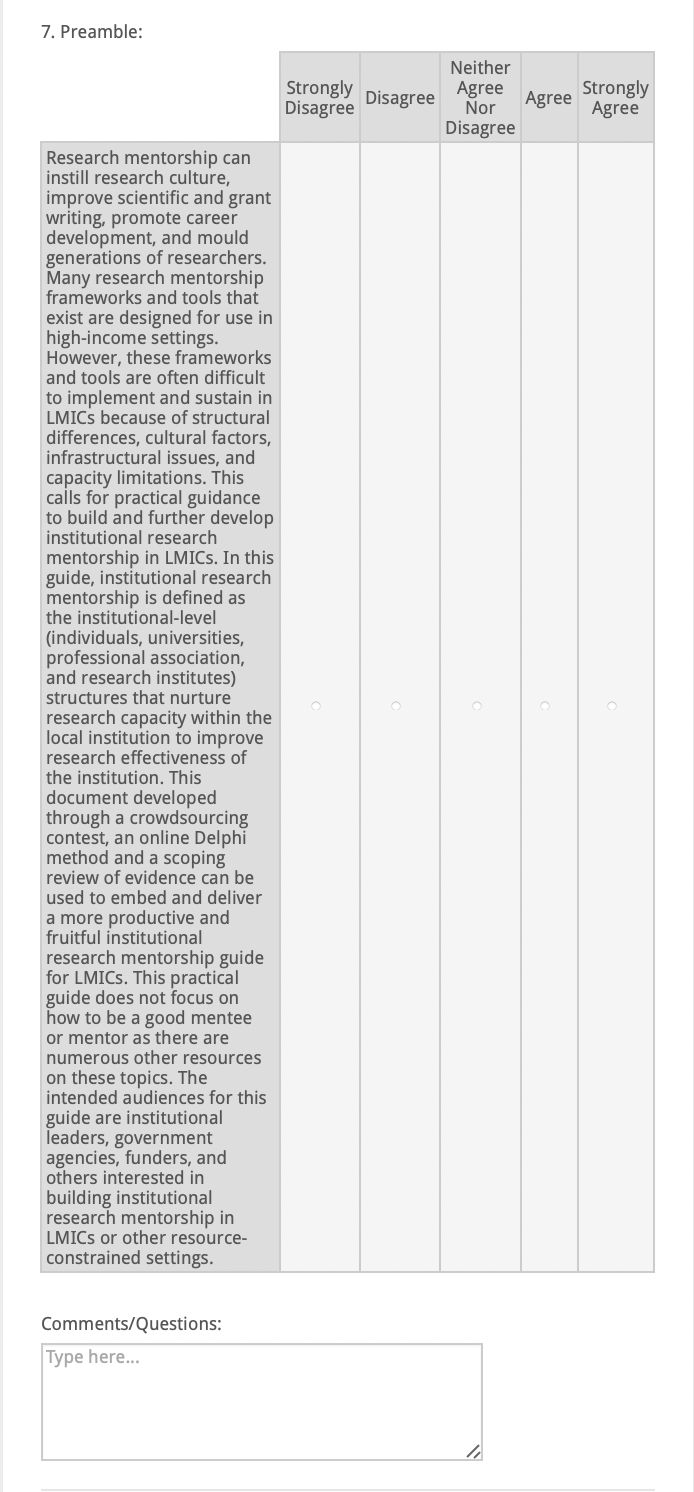

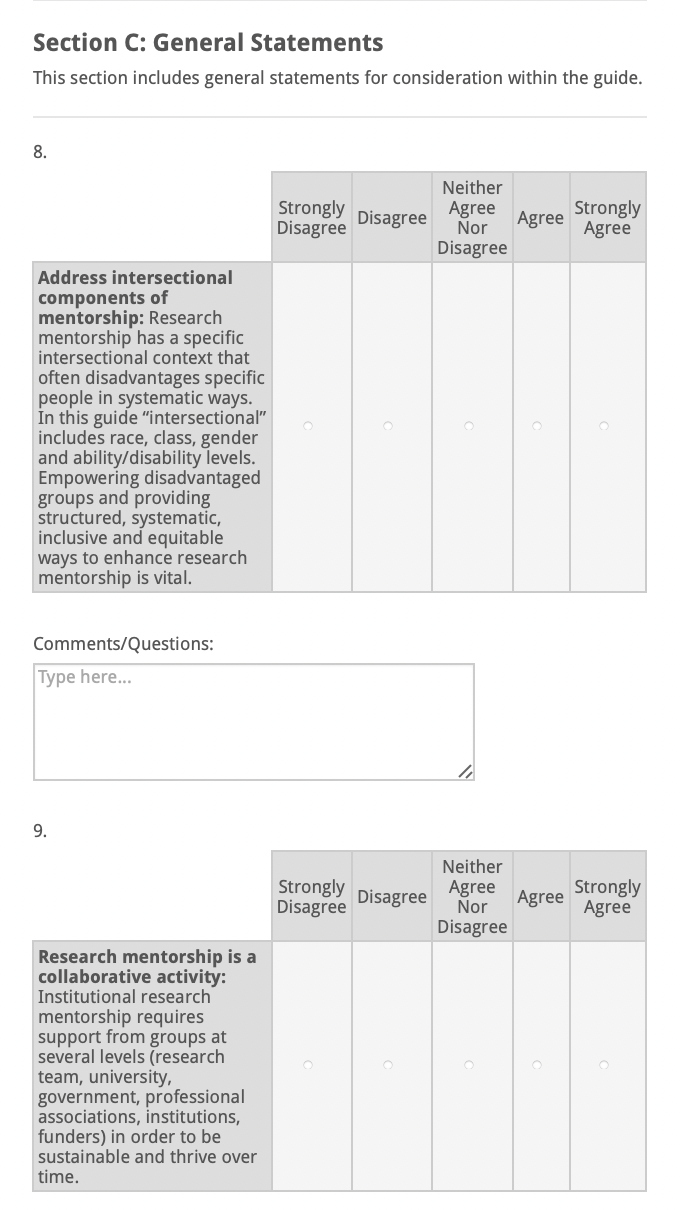

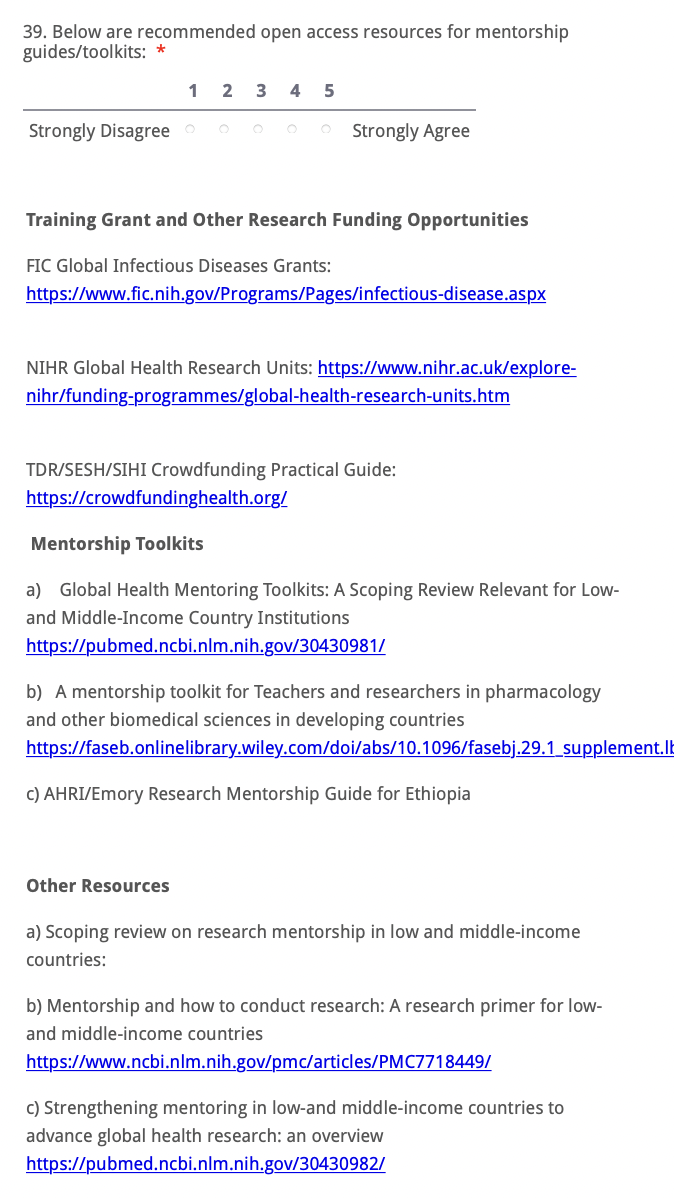

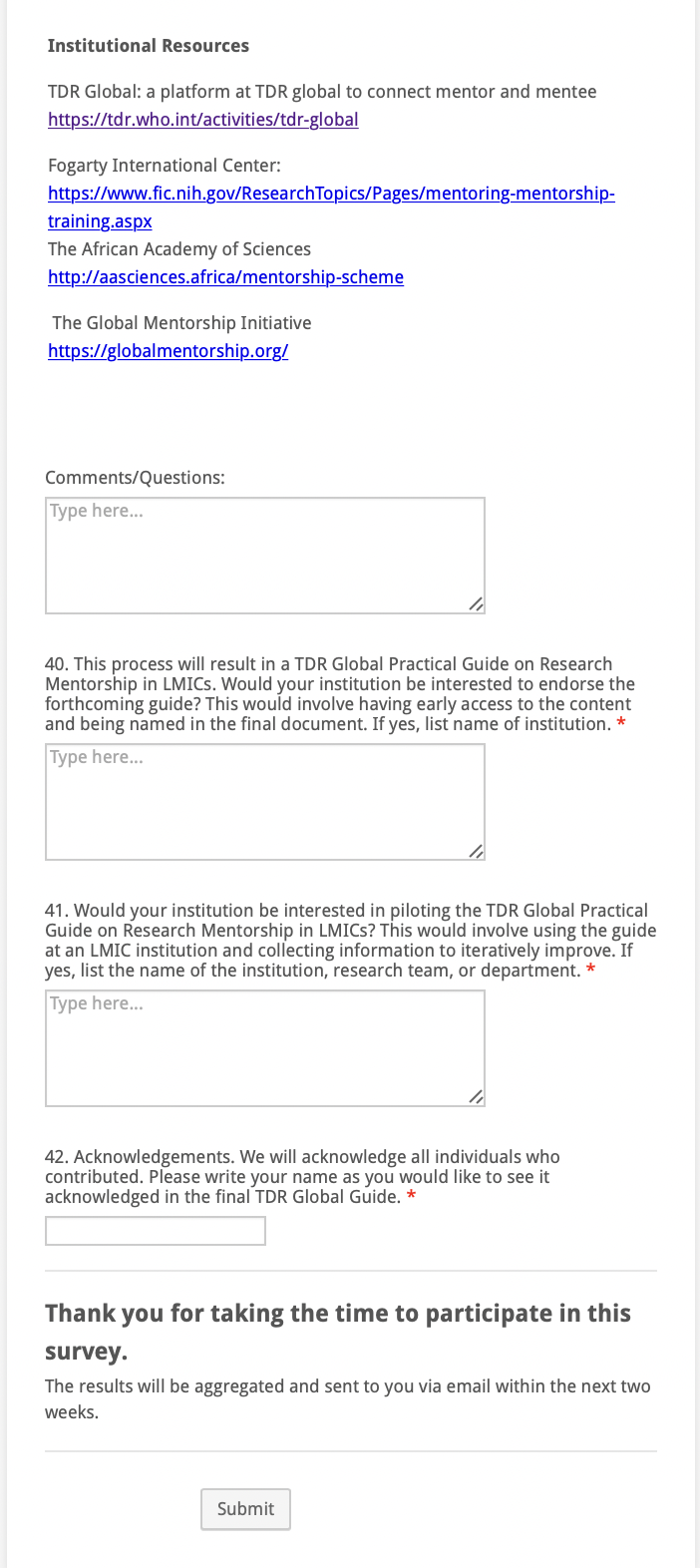

Supplement: S3 File — (DOCX) [file pone.0291816.s004.docx]
